# Metropolitan Hospital Sunday Fund

**Published:** 1919-08-16

**Authors:** 


### METROPOLITAN HOSPITAL SUNDAY FUND.

### Meeting of the Council.

A meeting of the Council of this Fund was held at the Mansion House on Wednesday, August 6, under the chairmanship of Mr. R. Holland-Martin.

Letters of regret at being unable to attend the meeting were received from (among others) the Bishop of Rochester, the Bishop of Islington, the Bishop of Southwark, the Archdeacon of Hampstead, the Chief Rabbi, the Archdeacon of London, the Archbishop of Westminster, Prebendary Proctor, and Dr. Fleming.

There were present Sir William Church, Sir John Bell, Sir C. C. Wakefield, Sir William Hale White, Mr. C. E. Spicer, Sir George Makins, Mr. J. R. Cooper, Rev. W. Hardy Harwood, Dr. Partridge, Mr. J. H. Buxton, Mr. Richard Peek, and Mr. John Robarts.

The Chairman proposed "That the Report of the Committee of Distribution for the year 1919 be, and the same is hereby, approved, and that the awards thereby recommended be paid forthwith." He said it would be seen that the amount collected this year totalled nearly £83,000. It was less by £2,600 than that received last year, but that was not so bad as it at first seemed, because last year's £85,000 included a special donation from the American Red Cross organisation. Apart from that special gift, therefore, the progress of the Fund from ordinary sources was very satisfactory, being about £2,500 more than for the previous twelve months. (Hear, hear.) He wished to make the further explanation that the amounts credited to the churches last year included a gift from Lady Strathcona of £2,000, whereas this year's contribution from her Ladyship of £3,000 was sent direct, so that the church receipts looked correspondingly less. He said he thought that though the splendid figure of last year had been even exceeded from collections, there was room for further improvement still. In the City a special appeal had been made on behalf of the Fund to business houses again, and numerous additional films had generously responded. The method yielded some striking increases, such as the following: St. Bartholomew-the-Great £23 last year, £76 this year; St. Mary Woolnoth £4 last year, £87 this year; St. Martin, Ludgate, £5 last year, this year £59; St. Michael, College Hill, £2, increased to £47. He hoped this source of income would be cultivated in the future more systematically and thoroughly than had yet been the case, and he appealed to members of the Council who were acquainted with the heads of business houses to use their influence in this direction.

The collections from the West End of London had somewhat fallen off, and three reasons were, in the main, given for this: the present heavy rate of taxation, the uncertainty as to what was likely to happen to the voluntary hospitals in the near future—*i.e.*, owing to the establishment of the Ministry of Health—and the unsettled state of national finance.

The County of London Red Cross Society collections resulted in an addition to the Fund of some £500.

Mr. J. R. Cooper seconded the motion. He considered that the Fund was still on the upward trend, and that the public had responded to the claims of the Fund in a most remarkable manner. The Report was an eminently satisfactory one. The resolution was carried.

### The Cost of Treatment in Cottage Hospitals.

Rev. W. Hardy Harwood moved "That the Committee be required to make necessary inquiries as to the cost of maintaining cottage hospitals, and report to the next Council meeting." He spoke specially of instances in which two cottage hospitals were within convenient reach of each other, neither of which had its beds anything like full at any one time, and yet there was a duplication of administration and motor transport, which would be avoided and greater economy practised if they were combined.

Sir William Church seconded, and the motion was unanimously adopted.

On the motion of the Chairman, seconded by Mr. C. E. Spicer, the best thanks of the Council were accorded to the Committee of Distribution for the labour and care they had bestowed on the recommendation of the awards to the several hospitals.

Sir Charles Wakefield proposed, in words of high appreciation, "That the Council's thanks be given to the Editors of various newspapers for the generous way in which they had pleaded the cause of the Fund."

Dr. Partridge seconded, and it was carried.

Sir John Bell proposed "That the cordial thanks of the Council be, and are hereby, tendered to the Right Hon. Sir Horace Brooks Marshall, Bart., P.C., Lord Mayor, who, as President and Treasurer, has devoted his time and valuable influence to the well-being of the Fund." He said Sir Horace had done yeoman service for this and other charitable funds, and he hoped this kindly influence would be continued.

Sir George Makins seconded, and it was carried.

### An Approach by the Charity Organisation Society.

The Secretary (Mr. Arnold James) read a letter which had been received from the Charity Organisation Society intimating the intention of that body to form a Standing Conference on Social Welfare, and inviting the Council of this Fund to nominate a representative to co-operate in such Conference.

The Chairman expressed his approval of the idea; the Charity Organisation Society was carrying on a very good work, and its usefulness must be enhanced by some such course as that suggested.

The Council agreed that the matter be referred to the General Purposes Committee to report.

A cordial vote of thanks to the Chairman terminated the proceedings.

### THE COMMITTEE OF DISTRIBUTION: REPORT AND AWARDS.

The Committee of Distribution held their first meeting on Monday, June 11, and then, and at subsequent meetings, examined the reports and income and expenditure accounts of the several institutions seeking to participate, and made their awards in conformity with the Laws of the Constitution.

The Committee beg to report that the total of the Fund on August 11 will amount to £83,000.

This year 228 institutions have applied to participate in the Fund, being ten less than in 1918, and the Committee now recommend the distribution of £82,462 13s. 8d. to the several hospitals, institutions, dispensaries and nursing associations, shown in the appendix hereto.

The Committee having reduced, or postponed the determination of the bases of award to eleven institutions, the governing bodies of the same were informed thereof in accordance with the Laws of the Constitution. Three deputations attended the Committee, and after hearing their

**Metropolitan Hospital Sunday Fund**—*(continued)*. several explanations, and those of the remainder having been received by letter, the bases of award were finally determined.

Your Commiteee recommend, in view of the high cost of treatment at many of the cottage hospitals, which appears to be mainly due to the relatively small proportion of occupied beds, that the Council should consider whether it should be proposed that where two or more of these institutions exist within a reasonable distance, they should take steps to amalgamate, with a view to more economical working.

Seven and a-half per cent. of the total sum available for distribution is appropriated to the purchase of surgical appliances during the ensuing year, and 2½ per cent. for district nursing associations.

In compliance with an order of the Council, statistics have been prepared as usual for the use of its members, showing the number of beds in hospitals, the cost of patients at hospitals and dispensaries, the proportionate expense of management to maintenance, and other information. A copy thereof has been sent to each member of the Council.

Horace B. Marshall, Lord Mayor, President and Treasurer.
R. Holland-Martin, Vice-President.
William S. Church, Chairman of Committee.
W. Hardy Harwood. G. H. Makins.
Somerleyton. Adrian Pollock.
John C. Bell. Marcus Samuel.
F. A. Bevan. W. Hale White.
C. C. Wakefield, John Henry Buxton, } Hon. Secs.

The Mansion House, August, 1919.

**Awards recommended for the year 1919.**

THIRTY-FIVE GENERAL HOSPITALS.

Charing Cross Hospital, £1,870 4s. 2d.; French Hospital, £135 2s. 6d.; Great Northern Central Hospital, £1,926 0s. 5d.; Guy's Hospital, £3,464 5s. 10d.; Hampstead and N.W. London Hospital, £1,075 2s. 6d.; Italian Hospital, £28 7s. 11d.; Kensington General Hospital, £160 11s. 8d.; King's College Hospital, £2,763 4s. 2d.; King Edward's, Ealing, £155 13s. 9d.; London Hospital, £10,011 19s. 7d.; London Homœopathic Hospital, £641 7s. 1d.; London Temperance Hospital, £709 17s. 11d.; Metropolitan Hospital, £1,376 14s. 2d.; Middlesex Hospital and Convalescent Home, £3,295 17s. 6d.; Mildmay Hospital, £302 11s. 3d.; Mildmay Memorial Hospital, £103 15s. 10d.; Miller Hospital and Royal Kent Dispensary, £839 2s. 11d.; Phillips' Memorial Homœopathic Hospital, £53 17s. 1d.; Poplar Hospital, £655 1s. 3d.; Prince of Wales' Hospital, £1,257 5s.; Royal Free Hospital, £1,971 1s. 3d.; St. Andrew's, Dollis Hill, £85 3s. 9d.; St. George's Hospital, £3,129 8s. 4d.; SS. John and Elizabeth Hospital, £159 12s. 1d.; St. John's Hospital, Lewisham, £254 11s. 8d.; St. Mary's Hospital, £2,082 13s. 9d.; St. Thomas's Hospital, £1,665 11s. 3d.; Seamen's Hospital Society, £953 14s. 2d.; University College Hospital, £1,722 7s. 1d.; Walthamstow, etc., Hospital, £201 14s. 2d.; Wandsworth, Bolingbroke Hospital, £505 5s.; West Ham Hospital, £1,298 7s. 6d.; West London Hospital, £1,864 6s. 8d.; Westminster Hospital, £1,611 14s. 2d.; Wimbledon Hospital, £55 16s. 3d.

SIX CHEST HOSPITALS.

City of London Hospital for Diseases of the Chest, Victoria Park, £1,397 5s. 5d.; Hospital for Consumption, Brompton, £2,343 2s. 11d.; Mount Vernon Consumption Hospital, £266 6s. 8d.; Royal Hospital for Diseases of the Chest, £286 17s. 11d.; Royal National Sanatorium, £39 3s. 4d.; Royal National Hospital, Ventnor, £293 15s.

TWENTY CHILDREN'S HOSPITALS.

Alexandra Hospital for Hip Disease, £104 15s. 5d.; Banstead Surgical Home, £34 5s. 5d.; Barnet Home Hospital, £31 6s. 8d.; Belgrave Hospital, £402 8s. 9d.; Cheyne Hospital for Incurable Children, £225 4s. 2d.; East London Hospital for Children, £1,001 13s. 9d.; Evelina Hospital for Sick Children, £352 10s.; Home for Incurable Children, £48 19s. 2d.; Home for Sick Children, Sydenham, £258 10s.; Hospital for Sick Children, £1,515 15s.; Infants Hospital, Westminster, £337 16s. 3d.; Kensington, for Children, and Dispensary, £99 17s. 6d.; Paddington Green Hospital for Children, £685 8s. 4d.; Queen's Hospital for Children, £1,818 6s. 3d.; Royal Waterloo Hospital for Children and Women, £539 10s. 5d.; St. Mary's Hospital, Plaistow, £355 8s. 9d.; St. Monica's Hospital, Brondesbury, £105 15s.; Victoria Hospital for Children, Chelsea, £736 6s. 8d.; Victoria Home, Margate, £29 7s. 6d.; Hospital for Hip Disease, Sevenoaks, £48 19s. 2d.;

NINE LYING-IN HOSPITALS.

British Home for Mothers and Babies, £24 9s. 7d.; City of London Maternity Hospital, £351 10s. 5d.; Clapham Maternity Hospital and Dispensary, £44 1s. 3d.; East End Mothers' Home, £149 16s. 3d.; General Lying-in Hospital, £369 2s. 11d.; Jewish Maternity, £94; Plaistow Maternity Hospital, £91 1s. 3d.; Queen Charlotte's Lying-in Hospital, £734 7s. 6d.; Salvation Army Lying-in Hospital, £62 13s. 4d.

SIX HOSPITALS FOR WOMEN.

Chelsea Hospital for Women, £373 1s. 3d.; Hospital for Women, £475 17s. 6d.; Grosvenor Hospital for Women, £193 17s. 6d.; Garrett Anderson Hospital, £649 3s. 9d.; Samaritan Free Hospital, £678 11s. 3d.; South London Hospital, £373 1s. 3d.

TWENTY-TWO OTHER SPECIAL HOSPITALS.

Middlesex Hospital, Cancer Wing, £322 2s. 11d.; London Fever Hospital, Islington, £244 15s. 10d.; St. Mark's Hospital for Fistula, £472 18s. 9d.; National Hospital for the Diseases of the Heart, £180 3s. 4d.; Female Lock Hospital, £518 19s. 2d.; Male Lock Hospital, £89 2s. 1d.; Hospital for Epilepsy, Paralysis, etc., £408 6s. 3d.; National Hospital for the Paralysed and Epileptic, £1,119 3s. 9d.; West End Hospital for Diseases of the Nervous System, £399 10s.; Central London Ophthalmic Hospital, £305 10s.; Royal Eye Hospital, £234 0s. 5d.; Royal London Ophthalmic Hospital, £1,179 17s. 11d.; Royal Westminster Ophthalmic Hospital, £221 5s. 10d.; Western Ophthalmic Hospital, £135 2s. 6d.; Royal National Orthopædic Hospital, £487 12s. 6d.; Royal Sea Bathing Hospital, Margate, £97 18s. 4d.; St. John's Hospital for Diseases of the Skin, £48 19s. 2d.; St. Peter's Hospital for Stone, £230 2s. 1d.; Central London Throat and Ear Hospital, £80 5s. 10d.; Throat Hospital, Golden Square, £166 9s. 2d.; Metropolitan Ear, Nose and Throat Hospital, £76 7s. 6d.; Royal Dental Hospital of London, £162 10s. 10d.

TWENTY-THREE CONVALESCENT HOSPITALS.

Metropolitan Convalescent Institution, Walton, £419 1s. 8d.; Metropolitan Convalescent Institution, Broadstairs, £48 19s. 2d.; Metropolitan Convalescent Institution, Bexhill, £721 12s. 11d.; All Saints' Convalescent Hospital, Eastbourne, £44 1s. 3d.; All Saints' Convalescent Home, St. Leonards, £27 8s. 4d.; Ascot Priory Convalescent Home, £39 3s. 4d.; Brentwood Convalescent Home for Children, £20 11s. 3d.; Chelsea Hospital for Women Convalescent Home, St. Leonards, £44 1s. 3d.; Hahnemann Convalescent Home, Bournemouth, £19 11s. 8d.; Hanwell Convalescent Home, £7 16s. 8d.; Hastings, Fairlight Sanatorium, £48 19s. 2d.; Herne Bay, Baldwin-Brown Convalescent Home, £48 19s. 2d.; Homœopathic Hospital Convalescent Home, £5 17s. 6d.; Mary Yolland Home, £26 8s. 9d.; Police Seaside Home, Brighton, £77 7s. 1d.; St. Andrew's Convalescent Home, Clewer, £48 19s. 2d.; St. Andrew's Convalescent Home, Folkestone, £146 17s. 6d.; St. John's Home, Kemp Town, £36 4s. 7d.; St. Joseph's Convalescent Home, Bournemouth, £14 13s. 9d.; St. Leonards-on-Sea Convalescent Home, £156 13s. 4d.; Eversfield, St. Leonards, £19 11s. 8d.; St. Mary's Convalescent Home, Birchington, £39 3s. 4d.; St. Mary's Convalescent Home, Broadstairs, £137 1s. 8d.

**Metropolitan Hospital Sunday Fund**—(*continued*).

TWENTY-FOUR COTTAGE HOSPITALS.

Acton Cottage Hospital, £122 7s. 11d.; Beckenham Cottage Hospital, £99 17s. 6d.; Blackheath and Charlton Cottage Hospital, £107 14s. 2d.; Brentwood Cottage Hospital, £44 1s. 3d.; Bromley, Kent, Cottage Hospital, £94; Canning Town Cottage Hospital, £171 7s. 1d.; Chislehurst, Sidcup and Cray Cottage Hospital, £118 9s. 7d.; Coldash Cottage Hospital, £61 13s. 9d.; East Ham Cottage Hospital, £30 7s. 1d.; Eltham Cottage Hospital, £62 13s. 4d.; Enfield Cottage Hospital, £109 13s. 4d.; Epsom and Ewell Cottage Hospital, £58 15s.; Hendon Cottage Hospital, £67 11s. 3d.; Hounslow Cottage Hospital, £79 6s. 3d.; Livingstone, Dartford Cottage Hospital, £164 10s.; Kingston Cottage Hospital, £73 8s. 9d.; Reigate and Redhill Hospital, £240 17s. 6d.; Sidcup Cottage Hospital, £44 1s. 3d.; Surbiton Cottage Hospital, £40 2s. 11d.; Teddington Cottage Hospital, £19 11s. 8d.; Tilbury (Passmore Edwards) Cottage Hospital, £48 19s. 2d.; Willesden Cottage Hospital, £65 12s. 1d.; Wimbledon (Nelson) Hospital, £142 19s. 2d.; Wood Green Cottage Hospital, £125 6s. 8d.

ELEVEN INSTITUTIONS.

Florence Nightingale Hospital for Invalid Gentlewomen, £205 12s. 6d.; St. Saviour's Hospital and Nursing Home, £113 11s. 8d.; Stoke Newington Home Hospital, £61 13s. 9d.; Firs Home, Bournemouth, £14 13s. 9d.; St. Columba's Hospital, £342 14s. 2d.; St. Luke's House, Pembridge Square, £137 1s. 8d.; Santa Claus Home, Highgate, £89 2s. 1d.; Royal Mineral Water Hospital, Bath, £24 9s. 7d.; Winifred House, Holloway, £62 13s. 4d.; Highgate Convalescent Home, £46 0s. 5d.; Holt Children's Sanatorium, £34 5s. 5d.

THIRTY-SEVEN DISPENSARIES.

Battersea Provident Dispensary, £133 3s. 4d.; Billingsgate Dispensary, £51 17s. 11d.; Brixton, etc., Dispensary, £47 19s. 7d.; Camberwell Provident Dispensary, £38 3s. 9d.; Childs Hill Provident Dispensary, £3 18s. 4d.; City Dispensary, £62 13s. 4d.; Clapham General and Provident Dispensary, £22 10s. 5d.; Eastern Dispensary, £26 8s. 9d.; East Dulwich Provident Dispensary, £47; Farringdon General Dispensary, £36 4s. 7d.; Finsbury Dispensary, £58 15s.; Forest Hill Provident Dispensary, £31 6s. 8d.; Greenwich Provident Dispensary, £23 10s.; Hampstead Provident Dispensary, £37 4s. 2d.; Islington Dispensary, £40 2s. 11d.; Islington Medical Mission, £41 2s. 6d.; Kilburn, Maida Vale and St. John's Wood Dispensary, £32 6s. 3d.; Kilburn Provident Medical Institution, £42 2s. 1d.; London Dispensary, Spitalfields, £12 14s. 7d.; London Medical Mission, Endell Street, £42 2s. 1d.; Margaret Street, for Consumption, £3 18s. 4d.; Metropolitan Dispensary, £26 8s. 9d.; Notting Hill Provident Dispensary, £19 11s. 8d.; Public Dispensary, £43 1s. 8d.; Queen Adelaide's Dispensary, £10 15s. 5d.; Royal General Dispensary, £22 10s. 5d.; Royal Pimlico Provident Dispensary, £19 11s. 8d.; St. George's, Hanover Square, Dispensary, £20 11s. 3d.; St. John's Wood Provident Dispensary, £29 7s. 6d.; St. Marylebone General Dispensary, £57 15s. 5d.; St. Pancras and Northern Dispensary, £59 14s. 7d.; Stamford Hill, Stoke Newington Dispensary, £44 1s. 3d.; Wandsworth Common Provident Dispensary, £6 17s. 1d.; Western Dispensary, £40 2s. 11d.; Western General Dispensary, £72 9s. 2d.; Westminster General Dispensary, £28 7s. 11d.; Woolwich Provident Dispensary, £30 7s. 1d.

THIRTY-TWO NURSING ASSOCIATIONS.

Belvedere, Abbey Wood, £10 15s.; Brixton, £43; Central St. Pancras, £53 15s.; Charlton and Blackheath, £10 15s.; Chelsea and Pimlico, £21 10s.; Hackney, £43; Hammersmith, £86; Hampstead, £32 5s.; Isleworth, £21 10s.; Kensington, £107 10s.; Kilburn, £10 15s.; Kingston, £32 5s.; Lambeth Road (Catholic), £21 10s.; Metropolitan (Bloomsbury), £75 5s.; St. Olave's (Bermondsey), £53 15s.; Paddington and Marylebone, £86; Plaistow, £86; Ponders End, Enfield, etc., £21 10s.; Rotherhithe, £21 10s.; Shoreditch, £86; Sick Room Helps Society, £10 15s.; Sidcup, £10 15s.; Silvertown, £21 10s.; South London (Battersea), £64 10s.; Southwark, £53 15s.; South Wimbledon, £32 5s.; Tottenham, £32 5s.; Westminster, £43; Woolwich, £64 10s.; East London, £150 10s.; North London, £75 5s.; Ranyard Nurses, £591 5s.

